# Abnormal serum microRNA profiles in tuberous sclerosis are normalized during treatment with everolimus: possible clinical implications

**DOI:** 10.1186/s13023-016-0512-1

**Published:** 2016-09-29

**Authors:** Joanna Trelinska, Wojciech Fendler, Iwona Dachowska, Katarzyna Kotulska, Sergiusz Jozwiak, Karolina Antosik, Piotr Gnys, Maciej Borowiec, Wojciech Mlynarski

**Affiliations:** 1 Department of Pediatrics, Oncology, Hematology and Diabetology, Medical University of Lodz, 36/50 Sporna Str., Lodz, 91-738 Poland; 2 Department of Biostatistics and Translational Medicine, Medical University of Lodz, Lodz, Poland; 3 Department of Neurology & Epileptology and Pediatric Rehabilitation, The Children’s Memorial Health Institute, Warsaw, Poland; 4 Department of Child Neurology, Medical University of Warsaw, Warsaw, Poland; 5 Department of Clinical Genetics, Medical University of Lodz, Lodz, Poland

**Keywords:** Tuberous sclerosis, mTOR inhibitor, everolimus, microRNA

## Abstract

**Background:**

Tuberous sclerosis (TSC) is a monogenic disease resulting from defects of the *TSC1* or *TSC2* genes, which encode the proteins forming hamartin-tuberin tumor suppressor complex, the mammalian target of rapamycin complex (mTOR). The mTOR pathway is constitutively activated in response to tuberin or hamartin defects. The mTOR pathway is also regulated by a multitude of epigenetic mechanisms, one of which is regulation by microRNA (miRNA) inhibition. This leads us to hypothesize that organ-level abnormalities of miRNA expression patterns are widespread in TSC. The aim of the study was to evaluate the serum profiles of miRNAs in patients with TSC and subependymal giant cell astrocytoma (SEGA) treated with mTOR inhibitor (everolimus).

**Methods:**

Serum microRNA profiling was performed in 10 TSC-patients before and three months after everolimus treatment, as well as in 10 sex- and age-matched healthy controls. MicroRNAs were profiled using qPCR panels (Exiqon).

**Results:**

Of 752 tested miRNAs, 11 showed statistically significant dysregulation in patients with TSC in comparison to controls. The following miRNAs were downregulated in TSC: miR-142-3p, miR-199a-5p, miR-142-5p and miR-136-5p; while miR-130a-3p, miR-378a-3p, miR-130b-3p, miR-192-5p, miR-25-3p, miR-215-5p and miR-222-3p were upregulated in TSC in comparison to the control group. After three months of everolimus treatment, mean dose 5.1 (2.6-9.7) mg/m^2^, seven miRNAs reached expression levels similar to healthy controls, with miR-142-3p and miR-136 showed significant increase over baseline levels in TSC patients. Moreover, miR-222-3p normalization due to treatment differed between patients with mutation in *TSC1* and *TSC2* gene.

**Conclusions:**

Activation of the mTOR pathway in TSC patients alters serum miRNA levels, which may be partially reversed by an mTOR inhibitor. This indicates the involvement of miRNA dysregulation in the pathogenesis of TSC, linking miRNA profiles with treatment efficiency.

**Electronic supplementary material:**

The online version of this article (doi:10.1186/s13023-016-0512-1) contains supplementary material, which is available to authorized users.

## Background

Tuberous sclerosis (TSC) is a monogenic disease resulting from defects of the tuberin (*TSC2*) or hamartin (*TSC1*) genes. The TSC phenotype encompasses the abnormal function of a range of organs and the formation of benign tumors, predominantly in the form of renal angiomyolipomas or subependymal giant cell astrocytoma (SEGA) in the brain [1]. The treatment of TSC has been considerably improved by the exploitation of the mTOR pathway (mammalian target of rapamycin), which is constitutively activated in response to tuberin or hamartin defects [2]. The mTOR signaling pathway is also regulated by a multitude of epigenetic mechanisms, one of which is regulation by microRNA (miRNA) inhibition [3–6], with miR-19 and miR-130a/b being major regulators of mTORC activity during early development [6], which suggests that organ-level abnormalities of miRNA expression patterns are widespread in TSC.

While tissue levels of miRNA expression are difficult, if not impossible, to study in humans, circulating miRNAs may be detected in the serum, making them applicable biomarkers of metabolic diseases [7] and various cancers [8]. Until now, however, no studies have been performed on the expression profiles of microRNAs present in the serum of patients with TSC. Therefore, the aim of the present study was to evaluate the serum miRNA profiles in patients with TSC before and after treatment with mTOR inhibitor (everolimus). To investigate whether abnormal serum profiles could be due to mTOR pathway activation, the present study examined whether treatment with an mTOR inhibitor (everolimus) would negate the alterations of the miRNA profile and thus pinpoint mTOR-dependent miRNAs in the serum.

## Methods

### Patients

The study group consisted of 10 children and young adults with TSCs of different genetic backgrounds and 10 healthy, age- and sex-matched controls. The TSC patients were described in our previous reports on response and complications of TSC treatment [9, 10]. The patients were recruited for the study between December 2011 and January 2014. The inclusion criteria were a positive diagnosis of TSC and treatment with everolimus (Votubia, Novartis, Germany). The indication for everolimus treatment was the presence of SEGA associated with TSC in patients who required therapeutic intervention but were not amenable to neurosurgery. From that cohort, only patients with serum samples drawn before the start of everolimus treatment were eligible for the present study, which resulted in the inclusion of 10 out of 18 individuals described in earlier reports [10].

Based on our previous experience we expected the most pronounced clinical response to everolimus therapy to occur within the first three months of treatment [9]. Therefore, the patients with TSC were evaluated at two time-points – before the initiation of treatment with everolimus and three months after its start to perform serum miRNA profiling. Serum levels of everolimus were monitored and titrated as described previously [10] aiming to maintain trough concentrations of everolimus higher than 5 ng/ml. After three months of mTOR inhibitor treatment with a titrated dose of 5.1 (2.6-9.7) mg/m^2^ daily, median everolimus serum concentration was 4.57 (1.50-12.50) in the blood sample used for miRNA profiling.

Written informed consent was given for everolimus treatment by the patients, or if the patient was under 16 years old, by their parent. The research was performed in accordance with the Declaration of Helsinki and has been approved by the Bioethics Committee of the Medical University of Lodz (RNN/113/14/KE).

### Molecular methods

For identification of *TSC1* and *TSC2* gene mutation, DNA was extracted from the blood samples using a QIAamp DNA Blood Mini Kit (Qiagen, Germany) following the manufacturer’s instructions. DNA samples were normalized to a final 5 ng/ul. A Trusight One sequencing kit (Illumina, San Diego, CA) was used to perform enrichment and final analysis of *TSC1* and *TSC2* genes. Each procedure was realized following the manufacturer’s instructions. Sanger DNA sequencing was used for validation of identified genetic variants.

Serum samples were obtained from patients with TSC and controls using standard vials with a coagulation activating agent (Becton-Dickinson, Franklin Lakes, NJ, USA). After clot formation, samples were centrifuged at 2000 rpm for 20 min. Afterwards, serum was collected into standard 0.6 ml Eppendorf vials and stored at −80 ° C until testing. A miRCURY™ RNA Isolation Kit- Biofluids (Exiqon, Copenhagen, Denmark) was used for miRNA isolation, according to the manufacturer’s protocol. Quantitative reverse transcription PCR of 752 different miRNAs was performed using the miRCURY LNA™ Universal RT microRNA PCR kit with ExiLENT SYBR Green according to the manufacturer’s instructions (Exiqon). Hemolysis was assessed using the miR-451/miR-23a ratio [11]. As a negative result was obtained for all of the samples, profiling of the whole dataset could proceed. Exiqon’s serum panels A and B were used for the profiling of circulating microRNAs.

### Statistical analysis

The mean expression of 56 miRNAs present in all of the studied samples was used for normalization of miRNA levels [12]. Only miRNA present in at least half of the samples from either group were considered for analysis. The formula for normalization was $dCq= average\;Cq\;\left(N=30\right)- assay\;Cq\;(sample)$. Higher dCq values thus indicated higher expression of a given miRNA. Cq values for specific miRNAs higher than 37 were filtered as absent calls. Initially, expression values for pretreatment TSC and control samples were compared using the Student’s t-test. The Benjamini-Hochberg procedure was used to evaluate false discovery rates (FDR). Post-treatment comparisons were compared with expression in controls using the Student’s t-test with Bonferroni adjustment for multiple comparisons to control for the family-wise error rate. The next step was to determine whether the change of any of the miRNAs that differed with the control group would show differences due to treatment with everolimus or mutated gene (*TSC1* or *TSC2*). To do so, a 2-way ANOVA procedure was used, which evaluated the impact of treatment, mutation and interaction of these factors. Adjusted *p* values below 0.05 and (where applicable) FDR < 0.05 were considered as statistically significant.

## Results

**Fig. 1:**
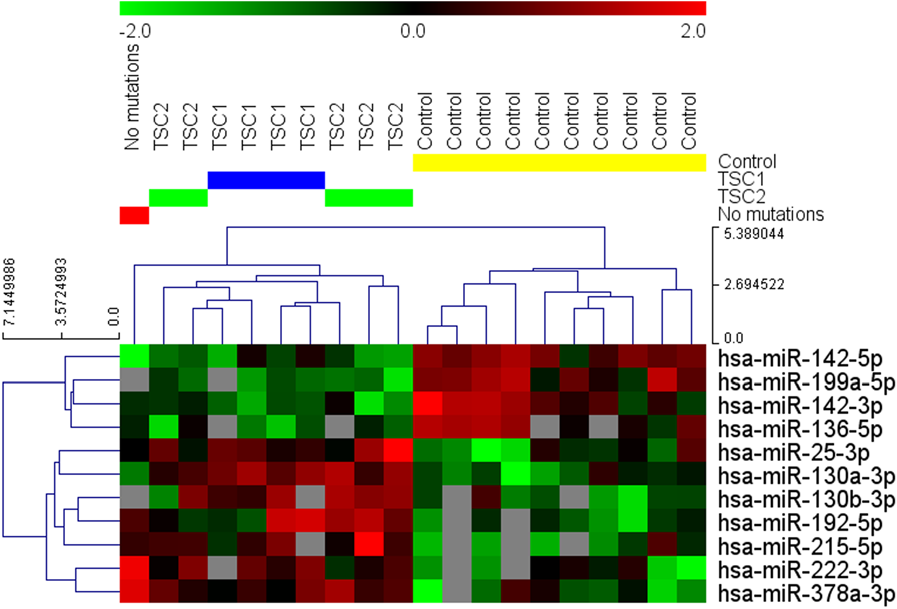
Expression scores of the 11 miRNAs that showed significant differences between the TSC and control groups. Higher dCq values represent higher expression as described in the Materials and Methods section. Expression values were normalized with one unit of the heatmap color range corresponding to one standard deviation of miRNA expression across the whole compared group. *Gray* panels represent lack of expression of a given miRNA in a specific sample

Before everolimus treatment the group of patients with TSC did not differ from the control group in terms of sex (6 M/4 F vs 4 M/6 F, p = 0.66) or age distribution (11.78 ± 4.44 vs 11.80 ± 4.66 years, p = 0.99). The clinical and genetic characteristics of patients with TSC were presented in Table 1. Causative mutations of the *TSC1* and *TSC2* genes are presented in an Additional file 1: Table S1. All 30 samples were eligible for profiling and comparison. Out of 752 tested microRNAs commonly detected in the human serum, 475 were detected in at least one sample. Out of that number, 136 were present in at least 50 % of TSC and control samples and were deemed eligible for analysis. Overall, 27 miRNAs differed significantly (p < 0.05) in unadjusted comparisons between TSC and control samples (Additional file 2: Table S2). Out of those miRNAs, 11 met the FDR criterion and were considered significant (Table 2). Raw data from miRNA profiling was presented as Additional file 3: Table S3. Four miRNAs were down-regulated in samples of patients with TSC in comparison to the controls: miR-142-3p, miR-199a-5p, miR-142-5p and miR-136-5p. The remaining 7 miRNAs (miR-130a-3p, miR-378a-3p, miR-130b-3p, miR-192-5p, miR-25-3p, miR-215-5p and miR-222-3p) showed higher expression in the TSC group than in the healthy controls. None of the miRNAs differed significantly depending on the type (mutations of *TSC1* vs *TSC2*) of TSC before the initiation of treatment with everolimus. However, the miRNA profiles of all patients with TSC, regardless of genetic background, clustered strongly and allowed for perfect discrimination with healthy individuals (Fig. 1).

**Table 1:** Clinical and genetic characteristics of the study group of patients with TSC

	Whole group (N = 10)	TSC2 (N = 5)	TSC1 (N = 4)	No mutation (N = 1)*
Sex (M/F)	6 / 4	2 / 3	4 / 0	1
Age (years)	11.78+/−4.44	10.32+/−5.41	13.25+/−3.61	13.17
Everolimus dose	5.95+/−2.08	6.99+/−1.77	4.56+/−2.1	6.3
Number of SEGA lesions				
• 1	7/10	2/5	4/4	1
• ≥2	3/10	3/5	0	0
• bilateral	3/10	3/5	0	0
Skin lesions:				
• Facial angiofibroma	9/10	5/5	3/4	1
• Fibrous cephalic plaque	3/10	2/5	0/4	1
• Hypomelanotic macules	7/10	5/5	1/4	1
• Shagreen patch	6/10	3/5	2/4	1
Other features:				
• Angiomyolipomas	5/10	4/5	0/4	1
• Multiple renal cysts	0	0	0	0
• Cardiac rhabdomyoma	5/10	3/5	2/4	0
• Retinal hamartomas	4/10	3/5	0	1
• Nonrenal hamartomas	1/10	0	1/4	0
Mental retardation	7/10	5/5	1/4	1
Epilepsy	8/10	5/5	2/4	1
Number of antiepileptic drugs				
• 1	1/10	0	1/4	0
• 2	4/10	3/5	0	1
• 3	3/10	2/5	1/4	0

*definite diagnosis of TSC was made according to the current clinical diagnostic criteria from The Tuberous Sclerosis Complex Diagnostic Criteria Update 2012

**Table 2:** Expression levels of miRNAs that showed significant difference between TSC and control groups. P-levels are calculated for a comparison of the whole TSC group with controls. Results for all miRNAs evaluated by the serum panels used for the profiling experiment are presented in Additional file 2: Table S2

	TSC2 (N = 5)	TSC1 (N = 4)	No mutation (N = 1)	All patients with TSC (N = 10)	Controls (N = 10)	Expression ratio TSC/Control	*P* value	Benjamini-Hochberg FDR
miR-142-5p	−1.71 ± 0.26	−1.35 ± 0.53	−2.49	−1.65 ± 0.49	−0.52 ± 0.34	0.46	0.0000	0.0010
miR-199a-5p	−2.93 ± 0.56	−2.84 ± 0.34	Undetected	−2.89 ± 0.47	−1.06 ± 0.73	0.28	0.0000	0.0010
miR-142-3p	0.84 ± 0.50	0.73 ± 0.23	1.06	0.82 ± 0.38	1.78 ± 0.57	0.52	0.0003	0.0146
miR-136-5p	−3.88 ± 0.85	−4.29 ± 0.51	−3.44	−3.98 ± 0.69	−2.35 ± 0.77	0.32	0.0005	0.0156
miR-130a-3p	−0.71 ± 0.33	−0.57 ± 0.18	−1.88	−0.77 ± 0.46	−1.70 ± 0.53	1.90	0.0006	0.0156
miR-378a-3p	−0.57 ± 0.33	−0.87 ± 0.40	0.35	−0.60 ± 0.48	−1.79 ± 0.75	2.28	0.0007	0.0157
miR-130b-3p	−4.16 ± 0.48	−4.16 ± 0.22	Undetected	−4.16 ± 0.38	−4.84 ± 0.32	1.60	0.0017	0.0287
miR-192-5p	−2.18 ± 0.55	−2.21 ± 0.86	−2.19	−2.20 ± 0.62	−3.14 ± 0.38	1.92	0.0017	0.0287
miR-25-3p	1.25 ± 0.56	1.09 ± 0.16	0.79	1.14 ± 0.41	0.44 ± 0.49	1.63	0.0027	0.0389
miR-215-5p	−3.18 ± 0.74	−3.58 ± 0.57	−3.51	−3.35 ± 0.63	−4.50 ± 0.64	2.21	0.0029	0.0389
miR-222-3p	−0.43 ± 0.44	−0.09 ± 0.30	0.92	−0.16 ± 0.56	−1.27 ± 0.74	2.16	0.0031	0.0389

During three months treatment with everolimus, significant reductions (>50 %) in SEGA volumes were recorded in 8/10 patients, while SEGA volume did not change in one patient and a 14.1 % increase in volume was noted in another. There was no difference in terms of % reduction in SEGA volume depending on the type of mutation (*TSC1* vs. *TSC2*).

**Fig. 2:**
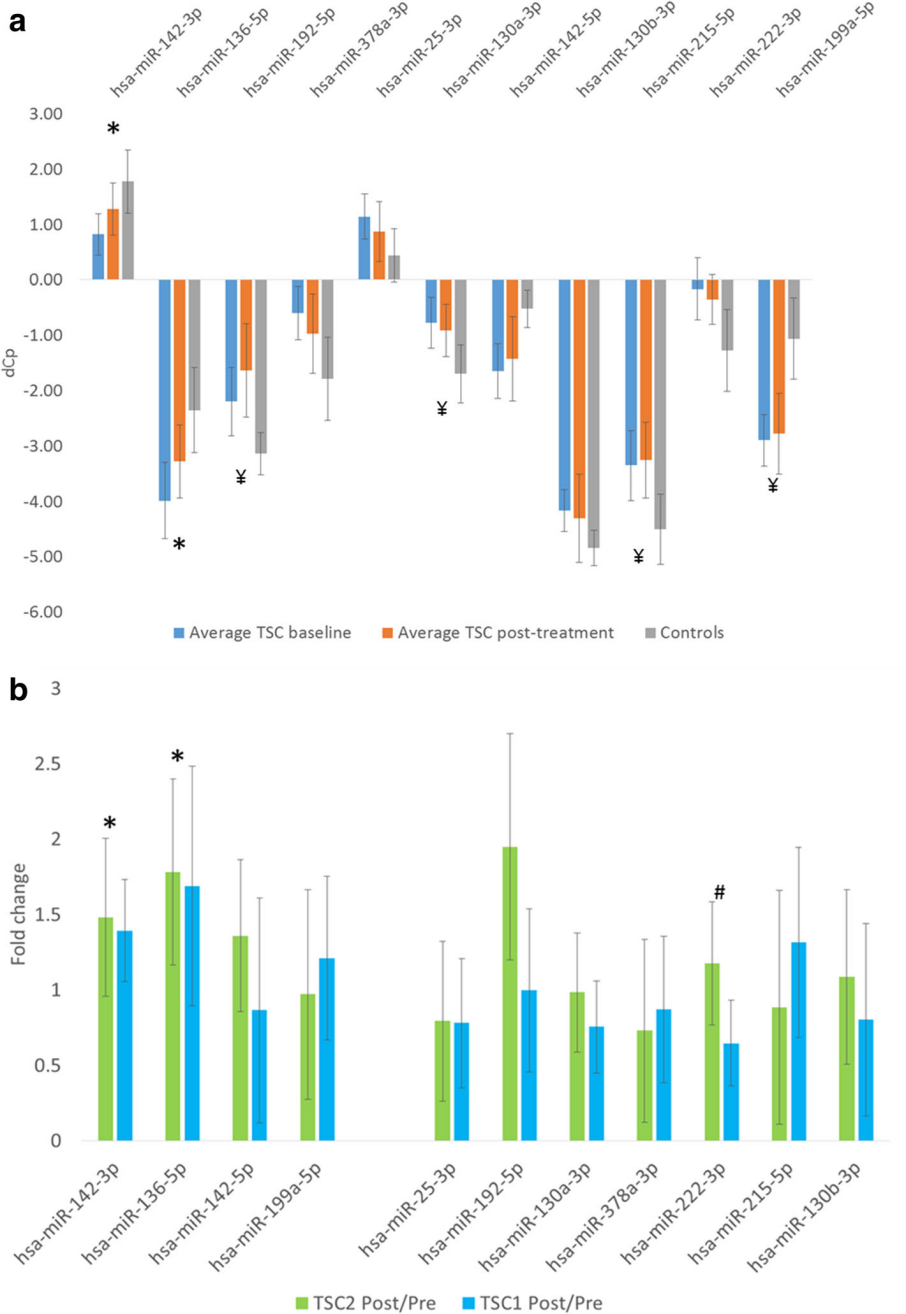
MiRNA expression level changes before and after treatment with Everolimus. **a** – average levels of specific miRNAs before and after treatment with everolimus and controls. In case of miR-136 and miR-142-3p, a significant increase in expression was observed after the introduction of therapy, making post-treatment expression levels closer to those observed in the control group. * - significant difference between pre- and post-everolimus expression levels and a lack of significant difference between post-everolimus and control levels, ¥ - significant difference between post-everolimus and control expression levels with non-significant change in paired pre- vs post-treatment expression levels. **b** – Interaction analysis of the impact of treatment with everolimus on miRNA expression levels. In case of mi-136 and miR-142-3p, significant increases were treatment-dependent but did not differ between the patients with TSC1 or TSC2. In case of miR222, a decrease (in this case, a change towards values observed in the control group) was observed only in patients with TSC1. * - significant for the pre/post treatment effect and non-significant for the treatment/TSC type interaction, # - significant for the treatment/TSC interaction and non-significant for the pre/post treatment effect in the whole group

Serum miRNA profiling performed three months after the introduction of everolimus treatment revealed statistically significant increases in the expression of miR-142-3 and miR-136-5p. In the case of miR-192, miR-130a, miR-215 (upregulated vs controls) and miR-199a (downregulated vs controls) the difference between pre- and post-treatment expression was not significant, but post-treatment expression levels were significantly different than those noted in the control group. For the remaining five miRNAs (miR-25, mi-378a, miR-142-5p, miR130b and miR-222), the difference in pre- and post-treatment expression as well as between post-treatment expression and controls was not statistically significant (Fig. 2a). In 10 out of the 11 differentially expressed miRNAs, the direction of miRNA expression change observed after everolimus treatment did not differ significantly in patients with mutations of *TSC1* and *TSC2* (Fig. 2b). However, while the expression of miR-222 decreased to a level similar to healthy controls in patients with mutations of *TSC1*, no differences from baseline values were noted in patients with mutations of *TSC2* (Fig. 2b). No association was found between clinical response to everolimus treatment measured as a SEGA volume change and miRNA levels (data not shown).

## Discussion

Our results show that TSC is a disease with considerably altered serum profile of miRNA. In addition, these alterations seem to be mTOR-dependent, as treatment with an mTOR inhibitor partially reversed the abnormalities. Furthermore, reaction to everolimus treatment resulted in TSC-type dependent changes of miRNA expression levels, hinting at a specific regulation of miR-222 and its role in altering the phenotypes of TSC1 and TSC2.

The differential expression of several miRNAs in patients with TSC was likely given the broad spectrum of clinical features observed in these diseases. While we are aware of phenotype variability observed in TSC patients, it was impossible to draw any conclusions from deregulated miRNA depending on the type of mutation or other clinical features, such as antiepileptic drug regimens, due to the small size of the study group. In this monogenic disease, the constitutively activated mTOR signaling leads to the formation of benign tumors and additional phenotypic traits such as seizures, intellectual disability and developmental delay [13, 14]. MiRNAs were shown to be associated with several clinical features of TSC, with miR-130a and miR-130b being linked to renal organogenesis [6]. In our study, both were significantly upregulated in comparison to the control group. The second-best characterized TSC-associated miRNA was miR-142, which has already been shown to inhibit the mTOR pathway [15]. In our dataset, levels of miR-142 were lower in the TSC group at baseline than in healthy controls and increased after everolimus treatment. One may speculate that everolimus acts as a direct inhibitor of mTORC1 protein complex by binding to FKBP12 protein and alternatively, indirectly stimulating miR-142 expression. Additionally, this reversible pattern of TSC-associated alteration of serum miRNA profile of miR-142 and miR-136 shows that serum levels of microRNAs may be also used as biomarkers of treatment efficiency and perhaps as predictive factors in rare disorders, in the same way as they are used in breast and prostate cancers [16, 17]. The fact that only miR-142-3p reverted to control values after treatment with everolimus may be due to its overall higher expression, involvement of miR-142-5p in intracellular regulatory mechanisms or differences in promoter sequences – further in-depth mechanistic studies would be needed to resolve the differences between miR-142-3p and -5p expression post-treatment.

The second notable finding of our results was the interaction between the genetic background of TSC and impact of everolimus treatment on serum level of miR-222. This miRNA was previously described as being associated with the KIT/AKT pathway in gastrointestinal stromal tumors [18] and peripheral nerve regeneration processes [19]. While no functional studies on the miR-222/AKT pathway associations were performed within this work, the differential everolimus-stimulated normalization of miR-222 expression observed in TSC1 and TSC2 patients is in line with the clinical observation that patients with TSC1 have a milder form of disease than TSC2. Whether miR-222 plays a role in shaping these phenotypes remains an open question. The absence of statistical differences present in miRNA expression profile, in *TSC1* vs *TSC2* mutation, or other clinical features, before everolimus treatment may be attributable to the limited number of patients with variable clinical characteristics at study entry.

Our results did not indicate any dysregulation of miR-21 reported in previous *in vitro* studies [20]; however, this may be explained by a number of mechanisms. Firstly, the cited work used a cellular model of a lymphangioleiomyomatosis with biallelic inactivation of *TSC2,* which was a different genetic defect than the one observed in our group. Furthermore, cellular changes of miRNA expression do not necessarily correspond to their serum levels, as the same miRNAs may originate from different cell types. A cellular model of miRNA expression patterns changing under direct rapamycin stimulation may not be thus directly translated onto serum levels. Moreover, Trindade et al. used a 132-miRNA panel and thus may not have picked out miRNAs present in the Exiqon 752-miR panel, which may have also contributed to the between-study discrepancy.

Our work does have several limitations mostly linked to the methodology of profiling and statistical power. Firstly, no attempt was made to profile exosome-bound miRNAs, but given that most of the miRNA present in the blood is contained in the exosomes [21] and were isolated during the total miRNA extraction protocol used in our study, it is unlikely that a significant bias could result from the analysis of free miRNA rather than only its exosome-bound fraction. Secondly, our investigation of TSC1/TSC2 differences and interactions with treatment effects had a very low statistical power.

Single point measurement after three months of mTOR inhibitor treatment may be considered as a limitation of the study. However, based on our experience and previous literature we expected the most pronounced clinical response to everolimus therapy to occur within the first three months of treatment [9, 22, 23]. Thus, we decided to study the change in miRNA profile during this time. Longer follow-ups, both clinical and molecular may be useful for further confirmation of our report.

Despite the low number of patients, the fact that our patients were examined before and after treatment with mTOR inhibitors make our preliminary results a valuable reference for future studies on the role of miRNA in TSC and mTOR signaling pathway abnormalities and their functional dependencies.

## Conclusions

Our results show for the first time that TSC is a disease which considerably alters serum miRNA levels and that changes of miR-142 and miR-136 may be reversed by treatment with an mTOR inhibitor, making their profiles potential indicators of treatment efficiency and hinting at their involvement in the pathogenesis of TSC.

## Additional files

Additional file 1: Table S1. Mutations responsible for the TSC phenotype in the study group. Reference sequences for *TSC2* (NM_000548.3; NP_000539.2) and *TSC1* (NM_000368.4; NP_000359.1) were used. (DOCX 17 kb) Additional file 2: Table S2. Univariate comparison results of miRNA expression in patients with tuberous sclerosis (TSC) before treatment and the control group. (XLSX 25 kb) Additional file 3: Table S3. Raw expression data (expressed as Ct) of the studied group. TSC - tuberous sclerosis. (XLSX 121 kb)

## Abbreviations

miRNA: microRNA
mTOR: mammalian target of rapamycin complex
SEGA: subependymal giant cell astrocytoma
TCS: Tuberous sclerosis complex
