# Supplementary material for: Abnormal serum microRNA profiles in tuberous sclerosis are normalized during treatment with everolimus: possible clinical implications
Source: Orphanet J Rare Dis. 2016 Sep 29;11:129. doi: 10.1186/s13023-016-0512-1 (PMC5041396; doi:10.1186/s13023-016-0512-1)
Supplement: Additional file 1: Table S1. — Mutations responsible for the TSC phenotype in the study group. Reference sequences for TSC2 (NM_000548.3; NP_000539.2) and TSC1 (NM_000368.4; NP_000359.1) were used. (DOCX 17 kb) [file 13023_2016_512_MOESM1_ESM.docx]

Additional file: Table S1 – Mutations responsible for the TSC phenotype in the study group. Reference sequences for *TSC2* (NM_000548.3; NP_000539.2) and *TSC1* (NM_000368.4; NP_000359.1) were used.

| Patient ID | Gene | Nucleotide change | Amino acid change |
| --- | --- | --- | --- |
| 725_201 | *TSC2* | c.1009G>A, | p.Glu337Lys |
| 738_201 | *TSC2* | c.2129G>C | p.Ala675Pro |
| 705_201 | *TSC2* | c.2354_2355+2delAGGT | splice_donor_variant |
| 752_201 | *TSC2* | c.5126 C>G | pPro1709Arg |
| 772_201 | *TSC2* | c.5227C>T | p.Arg1743Trp |
| 715_201 | *TSC1* | c.733C>T | p.Arg245Ter |
| 792_201 | *TSC1* | c.989_990 dupT | p.His253SerfsTer1 |
| 712_201 | *TSC1* | c.2071C>T | p.Arg692Ter |
| 713_201 | *TSC1* | c.2071C>T | p.Arg692Ter |
| 771_101 | *No mutation in *TSC1* and *TSC2* |  |  |

*Patient was studied for mutation in *TSC1* and *TSC2* gene using MLPA and NGS technology
